# Supplementary material for: Evaluation of Body Composition, Physical Activity, and Food Intake in Patients with Inborn Errors of Intermediary Metabolism
Source: Nutrients. 2021 Jun 20;13(6):2111. doi: 10.3390/nu13062111 (PMC8233825; doi:10.3390/nu13062111)
Supplement: Supplementary file 1 [file nutrients-13-02111-s001.zip › nutrients-1233985-supplementary.pdf]

Table S1. Mean body composition z-scores in patients and controls according to sex.

		Patients		Controls		
Anthropometric parameter (z-score)	Sex	N	Mean ± SD	N	Mean ± SD	*P*
Weight	F	57	0.33±1.09	50	0.44±0.76	0.534
	M	42	0.54±1.3	48	0.53±0.96	0.981
Height	F	57	-0.17±1.3	50	0.12±0.92	0.176
	M	42	-0.42±1.2	48	0.18±0.98	**0.008**
BMI	F	57	0.37±1.15	50	0.35±0.78	0.805
	M	42	0.81±1.5	48	0.48±1.01	0.164
Mid-arm circumference	F	57	-0.37±1.2	50	-0.4±1.02	0.884
	M	42	-0.24±1.2	48	-0.34±0.87	0.712
Waist circumference	F	57	-0.12±1.4	50	-0.56±1.2	**0.041**
	M	42	-0.03±1.3	48	-0.62±1.1	**0.046**
Hip circumference	F	57	-0.78±1.2	50	-0.86±0.8	0.687
	M	42	-0.49±1.2	48	-0.78±0.96	0.231
Biceps skinfold	F	57	0.57±1.7	50	0.55±1.3	0.957
	M	42	0.84±1.7	48	0.05±1.6	**0.026**
Triceps skinfold	F	57	-0.04±1.3	50	0.28±1.2	0.617
	M	42	0.23±1.2	48	-0.22±1.3	0.094
Subscapular skinfold	F	57	0.83±2.3	50	0.82±1.6	0.975
	M	42	1.28±2.5	48	0.37±1.9	0.062
Suprailiac skinfold	F	57	1.34±1.9	50	1.5±1.6	0.679
	M	42	1.4±1.7	48	0.54±1.6	**0.021**

F, females, M, males, BMI, body mass index, IEIM, inborn errors of intermediary metabolism. Differences considered significant at p <0.05.

Table S2. Mean dietary intake per day in patients and controls.

	Patients	Controls	
Dietary intake/day	Mean ± SD	Mean ± SD	*P*
Protein, total (g)	55.75±21.23	75.67±14.61	**0.000**
Energy, protein (Kcal)	216.19±83.63	302.59±58.57	**0.000**
Energy, protein %	13.95±4.65	20.06±3.77	**0.000**
Fat, total (g)	47.96±20.21	47.73±10.86	0.802
Energy, fat (Kcal)	418.37±158.69	430.40±97.42	0.628
Energy, fat %	26.91±7.43	28.08±5.22	0.290
CH, total (g)	234.57±119.76	201.79±39.26	**0.013**
Energy, CH (Kcal)	893.84±265.69	808.76±157.07	**0.008**
Energy, CH %	88.34±5.68	52.46±5.81	0.093
Energy, total (Kcal)	1566.93±399.72	1540.83±224.77	0.487
Energy (Kcal/kg)	40.99±17.06	43.09±19.49	0.489
Protein, total (g/kg)	1.41±0.65	2.08±0.89	**0.000**
Cholesterol (mg)	172.97±104.99	179.86±102.62	0.647
Sodium (mg)	1111.54±346.58	1397.71±593.01	**0.000**
Potassium (mg)	1814.71±503.78	1782.63±598.12	0.688

Calcium (mg)	717.46±231.69	673.46±325.80	0.284
Magnesium (mg)	190.26±74.40	182.71±86.32	0.517
Phosphorus (mg)	879.07±273.92	874.15±323.69	0.117
Iron (mg)	11.84±7.22	10.17±7.35	0.910
Fluor (µg)	10.96±23.01	5.21±14.56	**0.042**
Selenium (µg)	64.57±29.67	66.91±34.97	0.617
Zinc (mg)	7.18±3.42	6.88±3.57	0.557
Folate (µg)	283.30±155.27	226.13±165.30	**0.015**
Vitamin B12 (µg)	5.91±6.78	6.61±15.41	0.685
Vitamin A (µg)	527.28±296.93	440.7±301.52	**0.047**
Vitamin D (µg)	1.49±0.82	1.56±1.23	0.624
Vitamin K (mg)	71.66±63.73	75.77±94.87	0.724
Vitamin E (mg)	4.51±3.52	4.16±3.47	0.491

CH, carbohydrate. Differences considered significant at p<0.05.

Table S3. Mean values in biochemical blood analysis in IEIM patients and controls.

	**Patients**	**Controls**	
Blood variable	Mean± SD	Mean± SD	*P*
Prealbumin (mg/dL)	22.98±5.59	22.66±4.56	0.667
RBP (mg/dL)	3.51±0.97	3.72±2.35	0.421
Protein, total (g/dL)	7.27±0.35	7.21±0.37	0.283
Albumin (g/dL)	4.64±0.214	4.69±0.207	0.798
Calcium (mg/dL)	9.77±0.33	9.76±0.29	0.771
Zinc (µg/dl)	101.69±17.63	103.3±12.44	0.468
Selenium (µg/L)	76.37±18.01	85.46±8.01	**0.000**
Iron (µg/dL)	89.8±33.19	89.55±34.81	0.967
Ferritin (ng/mL)	38.01±27.64	40.48±38.93	0.620
Transferrin (mg/dL)	291.08±43.01	264.34±46.96	0.685
Vitamin A (mg/dL)	0.39±0.14	0.36±0.11	0.325
Vitamin D (ng/mL)	23.86±11.58	22.33±6.8	0.272
Vitamin E (mg/dL)	1.69±2.01	4.38±4.81	**0.003**
Vitamin k (ng/dL)	0.91±1.45	0.32±0.26	**0.003**
Folate (ng/mL)	17.82±11.41	8.99±3.79	**0.000**
Vitamin B12 (pg/mL)	1621.4±8030.14	577.95±221.79	**0.046**
Cholesterol (mg/dL)	157.86±26.44	165.6±27.12	**0.047**
TG (mg/dL)	84.2±43.39	59.77±27.57	**0.000**
HDL (mg/dL)	56.92±51.51	57.71±11.55	0.888
LDL (mg/dL)	87.78±19.65	107.88±110.95	0.126

HDL, high density lipoprotein, LDL, low density lipoprotein, RBP, retinol binding protein, TG, triglyceride. Differences considered significant at p<0.05.
